# Dominant side in single-leg stance stability during floor oscillations at various frequencies

**DOI:** 10.1186/1880-6805-33-25

**Published:** 2014-08-15

**Authors:** Takeo Kiyota, Katsuo Fujiwara

**Affiliations:** 1 Department of Psychology, Faculty of Humanities, Sapporo International University, 4-1-4-1 Kiyota, Kiyota-ku, Sapporo 004-8602, Japan; 2 Department of Human Movement and Health, Graduate School of Medical Science, Kanazawa University, 13-1 Takara-machi, Kanazawa 920-8640, Japan

**Keywords:** Dynamic balance, Lateral dominance, Single-leg stance, Static balance

## Abstract

**Background:**

We investigated lateral dominance in the postural stability of single-leg stance with anteroposterior floor oscillations at various frequencies.

**Methods:**

Thirty adults maintained a single-leg stance on a force platform for 20 seconds per trial. Trials were performed with no oscillation (static condition) and with anteroposterior floor oscillations (2.5-cm amplitude) at six frequencies: 0.25, 0.5, 0.75, 1.0, 1.25 and 1.5 Hz (dynamic condition). A set of three trials was performed on each leg in each oscillation frequency in random order. The mean speed of the center of pressure in the anteroposterior direction (CoP_ap_) was calculated as an index of postural stability, and frequency analysis of CoP_ap_ sway was performed. Footedness for carrying out mobilizing activities was assessed with a questionnaire.

**Results:**

CoP_ap_ speed exponentially increased as oscillation frequency increased in both legs. The frequency analysis of CoP_ap_ showed a peak <0.3 Hz at no oscillation. The frequency components at 0.25-Hz oscillation included common components with no oscillation and those at 1.5-Hz oscillation showed the maximum amplitude among all conditions. Postural stability showed no significant difference between left- and right-leg stance at no oscillation and oscillations ≤1.25 Hz, but at 1.5-Hz oscillation was significantly higher in the right-leg stance than in the left-leg stance. For the lateral dominance of postural stability at individual levels, the lateral difference in postural stability at no oscillation was positively correlated with that at 0.25-Hz oscillation (*r* = 0.51) and negatively correlated with that at 1.5-Hz oscillation (*r* = -0.53). For 70% of subjects, the dominant side of postural stability was different at no oscillation and 1.5-Hz oscillation. In the subjects with left- or right-side dominance at no oscillation, 94% or 38% changed their dominant side at 1.5-Hz oscillation, with a significant difference between these percentages. In the 1.5-Hz oscillation, 73% of subjects had concordance between the dominant side of postural stability and that of mobilizing footedness.

**Conclusion:**

In static conditions, there was no lateral dominance of stability during single-leg stance. At 1.5-Hz oscillation, the highest frequency, right-side dominance of postural stability was recognized. Functional role in supporting leg may be divided between left and right legs according to the change of balance condition from static to dynamic.

## Background

In humans, the lower limbs play an important role in providing postural support for maintaining standing posture. The upper limbs are not involved in supporting body weight unless the subject stands or walks with a cane or crutches and they thus assume a role in manipulation of objects. In a general sense, the upper and lower limbs are anatomically symmetrical across the sagittal plane of the body, but one of the bilateral limbs is preferentially used. In humans, this characteristic is called *lateral dominance*[1,2]. Approximately 90% of adults exhibit right-side dominance in manipulative functions of the upper limb [3] and in mobilizing functions of the lower limb, such as when kicking or juggling a ball [4-6]. However, when the lower limb is used as a postural support during single-leg stance, there is no clear lateral dominance in postural stability, even though the dominant side in maintaining stability is often shown at the individual level [7,8]. These findings regarding the postural support function have been assessed primarily by the measure of fluctuation of center of pressure (CoP) during stance on a stable surface (that is, a static balance condition).

However, in activities of daily living, including sports, it is more important to maintain postural stability under unstable balance conditions, when external forces besides gravity force act as perturbations or the whole body moves in space (that is, dynamic balance conditions). In addition, the control strategy for maintaining single-leg standing posture on vibrating (swaying) surfaces is sometimes needed in particular cases (for example, working in vehicles or aircraft). The lateral dominance of postural stability in dynamic conditions has been investigated using a movable semicircle seesaw on a force platform [9-11], but no significant lateral difference was found. For maintaining a stable standing posture against gravity force, the body alignment should be controlled appropriately and CoP position should be kept within the base of support [12]. On this seesaw, especially, CoP position must remain on the supporting point. However, this point does not move by external force; rather, it moves by the person’s involuntary and irregular body sway on the seesaw. Therefore, it would be difficult to anticipate its movement and set CoP on the supporting point. This would lead to large intraindividual variability in postural stability during single-leg stance on this seesaw, resulting in no significant lateral dominance. On the other hand, in the case of a periodic floor oscillation, used as a postural perturbation, it is easy to set CoP at a certain range within the base of support, because subjects can easily anticipate the disturbance timing for its periodicity [13]. Therefore, the postural stability on the oscillating floor has high reproducibility [13]. In addition, the periodical floor oscillation is excellent in the quantification of stimulus intensity because the acceleration of disturbance changes according to the oscillation frequency [14]. Mean speed of CoP in the anteroposterior direction (CoP_ap_) has been used to evaluate postural stability during this perturbation [15].

Fujiwara *et al*. [16] investigated the spectrum of postural sway during bipedal stance on a floor that oscillated between 0.1 Hz (a relatively static balance condition) and 1.5 Hz (a dynamic balance condition) [16]. At low-frequency oscillation (0.1 Hz), low-frequency components similar to those during quiet standing posture (below 0.5 Hz) were observed, as well as a peak component at the oscillation frequency. On the other hand, at higher-frequency oscillations (≥0.5 Hz), the peak amplitude at the oscillation frequency increased remarkably, and the low-frequency components were also observed. These findings indicate difference between postural controls during bipedal stance at low-frequency and high-frequency oscillations, specifically static and dynamic balance conditions, respectively. However, postural stability and postural control during single-leg stance at various frequency oscillations has not been investigated. The analyses of mean speed and spectrum of CoP_ap_ will reveal the difference of postural stability during single-leg stance between static and balance conditions. We predicted that by also using the floor oscillation no lateral difference of postural stability would be found in relatively static conditions (that is, low-frequency conditions). On the other hand, at high-frequency oscillation, when dynamic postural control is required, a clear lateral difference will be found. Postural stability in single-leg stance during voluntary step initiation has reportedly been better in the dominant foot than in the nondominant foot of mobilizing function [17]. Therefore, we predicted that at high-frequency oscillations, postural stability would be higher in the mobilizing dominant foot (that is, the right foot) than in the nondominant foot (that is, the left foot).

In this study, we investigated lateral dominance in postural stability during single-leg stance with anteroposterior floor oscillations at various frequencies. The following were our working hypotheses:

1. With low-frequency oscillations, similar to the static balance condition, no significant lateral difference would be shown, whereas, on an individual level, the dominant side of postural stability would be the same as that in static balance.

2. With increases in oscillation frequency, the postural stability of the dominant side for mobilizing functions would become greater than those of the nondominant side.

## Methods

### Subjects

Subjects were 30 healthy young adults (17 men, 13 women). Mean (standard deviation) age, height, weight, right-foot length and left-foot length were 21.6 (3.0) years, 166.6 (7.6) cm, 59.3 (7.9) kg, 24.6 (1.5) cm and 24.6 (1.5) cm, respectively. Written informed consent was obtained from all subjects in accordance with the Declaration of Helsinki following an explanation of the experimental protocols. The study and the study protocol were approved by the Kanazawa University Ethics Committee.

### Apparatus and data recording

**Figure 1:**
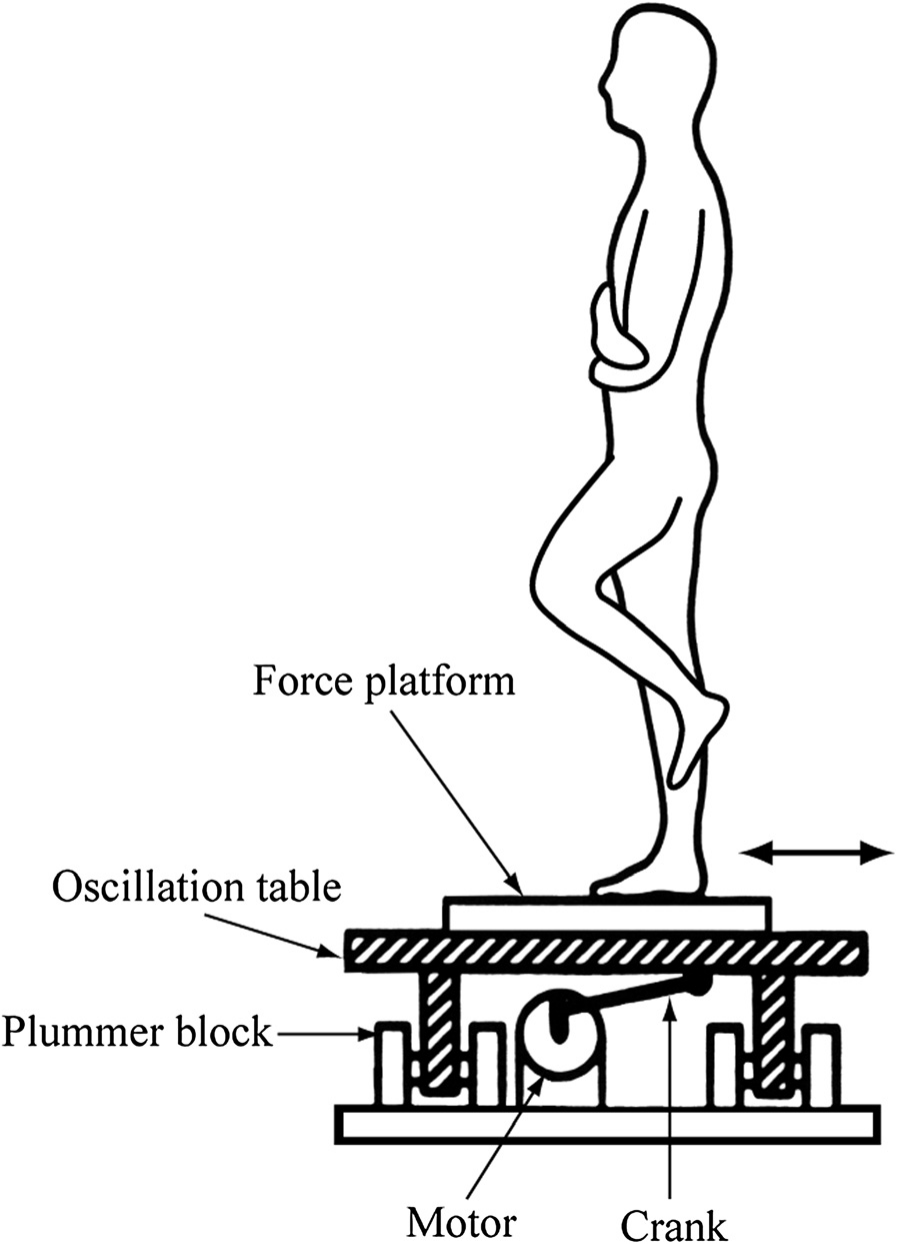
**Experimental setup.** Floor oscillation and center of pressure were recorded during single-leg stance.

A force platform (50 cm long and 50 cm wide, S110; Patella, Tokyo, Japan) consisting of three load cells was used to record CoP_ap_. CoP_ap_ position was calculated based on force data from the three load cells and distance between load cells. The formulae for these calculations are described in detail in our previous study [18]. An oscillation table (PW0198; Electric Control Group, Tokyo, Japan) with an attached force platform oscillated sinusoidally in the anteroposterior direction with a 2.5-cm amplitude (Figure 1). Height from ground to the platform surface was 32 cm. Table oscillation was detected using a linear position sensor (LP10; Midori, Chikuma, Japan), and oscillation frequency was measured using a frequency counter (TR-5822; Advantest, Tokyo, Japan). A visual target (10-mm diameter) was placed 1.5 m in front of the force platform at eye level. All electrical signals were sent to a computer (Dimension E521; Dell Japan, Kawasaki, Japan) via an A/D converter (ADA16-32/2(CB)F; Contec, Osaka, Japan) with a 1,000-Hz sampling rate and 16-bit resolution.

### Procedure

In order to adapt to the floor oscillation at each frequency (0.25, 0.5, 0.75, 1.0, 1.25 and 1.5 Hz), subjects maintained a bipedal standing posture on the oscillating platform with bare feet 10 cm apart and parallel, and with eyes open and arms folded across the chest, for 60 seconds before performing a single-leg stance. During single-leg stance, subjects stood on either the left or right leg for 20 seconds with the medial malleolus of the lifted leg touching the muscle belly of gastrocnemius medialis of the supporting leg, eyes open and arms folded across the chest. A single adaptation trial during bipedal standing was performed at each frequency. Three adaptation trials during single-leg stance were performed on each leg. When the CoP_ap_ speed in the third trials decreased by >10% from the value of the second trial, additional trials were performed in order to complete the adaptation process. Trials were stopped when the percentage change of CoP_ap_ speed between any two consecutive additional trials dropped below 10%. Subjects were instructed not to intentionally flex their knees or trunk during the oscillation. Adaptation trials were performed from the highest to the lowest oscillation frequency (that is, from 1.5 to 0.25 Hz) with a 60-second seated rest between trials.Next, experimental trials were performed for 20 seconds in the no-oscillation and oscillation conditions (Figure 1). Subjects who were instructed to gaze at the visual target and to stand as still as possible gave the experimenter a verbal signal within 5 seconds of the start of the trial if they were able to maintain single-leg stance. If the verbal signal was given more than 5 seconds after the start of the trial, the trial was stopped and restarted after 60 seconds of seated rest. First, six trials (three trials per leg) were performed in the no-oscillation condition. Subsequently, a set of three trials was performed on each leg in each oscillation frequency in random order. Half of the subjects were measured in the order of no-oscillation and oscillation conditions, and the other half were measured in reverse order. Subjects had a 60-second seated rest between trials. In the oscillation condition, subjects were supported by the experimenter for the first 2 seconds of the oscillation. After that, support was not provided unless the subject appeared to be in danger of falling.

### Footedness questionnaire

The preferred foot for manipulating an object (mobilizing function of footedness) was assessed using the Waterloo Footedness Questionnaire – Revised [19]. The questions were as follows: (1) Which foot would you use to kick a stationary ball at a target straight in front of you? (2) Which foot would you use to smooth sand at the beach? (3) Which foot would you use to stomp on a fast-moving bug? (4) If you wanted to pick up a marble with your toes, which foot would you use? (5) Which foot would you use to help push a shovel into the ground? Responses of left-always, left-usually, equal, right-usually, and right-always were scored on a scale of -2 to +2. This gave a range of scores from -10 for the most left-footed to +10 for the most right-footed. Scores from -10 to -5, -4 to +4, and +5 to +10 were taken to indicate left-footedness, mixed-footedness, and right-footedness, respectively.

### Data analysis

The electrical CoP_ap_ signal was transmitted to a computer (Epson, PC-286LS, Suwa, Japan) via an A/D converter (I/O-data, PIO9045, Kanazawa, Japan) with 20-Hz sampling rate and 12-bit resolution. These CoP_ap_ signals were smoothed using formula A, and mean speed of CoP_ap_ (in millimeters per second) was then calculated with formula B:

Formula A (smoothing anterior-posterior CoP_ap_ displacement):

$$\begin{array}{c} Y_{n}=(-3\times X_{n-2}+12\times X_{n-1}+17\times X_{n}+12\\ \;\times X_{n+1}-3\times X_{n+2})/35 \end{array}$$

(*X*_n_: sampling value; *Y*_n_: nth weighted average)

Formula B (calculation of mean speed of CoP_ap_):

$$\text{Co}P_{\text{ap}}\text{speed}=\frac{20}{N-1}\overset{N-1}{\underset{i=1}{\sum }}\left|y_{i+1}-y_{i}\right|$$

(*N*: sampling number, *y*_i_: sampling value)

It has previously been reported that, in a rigid body model, the mean speed of CoP_ap_ is influenced by the height of the center of mass during a constant frequency oscillation [13]. Therefore, CoP_ap_ speed measurement values were corrected for subject height by Formula C:

Formula C (normalizing CoP_ap_ speed for height):

$$\begin{array}{c} \text{Normalized}\;\text{Co}P_{\text{ap}}\text{speed}\;=\text{measured}\;\text{Co}P_{\text{ap}}\text{speed}\\ \;\times 100\;\left(\text{cm}\right)\;/\;\text{height}\;\left(\text{cm}\right) \end{array}$$

In each condition, the mean value of three trials was calculated. The lateral difference in postural stability was quantified as the difference in CoP_ap_ speed between the right and left sides, and the difference was expressed relative to the mean value of the right and left sides. A positive lateral difference indicated left-side dominance, and a negative lateral difference indicated right-side dominance.

For the frequency analysis of CoP_ap_ sway, fast Fourier transformation was performed on the full 20 seconds of data with 0.061-Hz resolution and a Hanning window. The amplitude of the frequency spectrum was measured between 0 and 2 Hz, and a spectrum peak at oscillation frequency, and the low-frequency component between 0 and 0.5 Hz was calculated. In a previous study, the spectrum peak at the oscillation frequency was used as an index of the effects of balance training in young adults [13]. In addition, it was previously reported that a low-frequency component at 0 to 0.5 Hz in body sway accounted for visuovestibular regulation [20,21]. The spectrum in present study was calculated using BIMUTAS II software (Kissei Comtec, Matsumoto, Japan).

If subjects moved their feet, they were supported by the experimenter, or if the medial malleolus of the lifted leg became separated from the supporting leg during the oscillation trial, the data corresponding to the interrupting event and in the 3 seconds after recovery of a stable posture were discarded. Thirteen subjects (43.3%) experienced at least one of these events, and the shortest trial across all subjects was 15.3 seconds. For the right foot, the mean (standard deviation) duration of data used for the analysis was 19.9 (0.38) seconds in the no-oscillation condition and 19.8 (0.38), 19.9 (0.38), 19.9 (0.57), 19.5 (1.1), 19.8 (0.62) and 19.7 (0.74) seconds, respectively, for oscillations at 0.25, 0.5, 0.75, 1.0, 1.25 and 1.5 Hz, respectively. For the left foot, the mean duration of data used for the analysis was 19.9 (0.30) seconds in the no-oscillation condition and 19.9 (0.22), 19.7 (0.62), 19.9 (0.54), 19.7 (0.74), 19.8 (0.51) and 19.8 (0.70) seconds, respectively, for oscillations at 0.25, 0.5, 0.75, 1.0, 1.25 and 1.5 Hz, respectively. There were no significant differences between right and left sides or across conditions in the duration of data used for the analysis.

### Statistical analysis

Shapiro-Wilks tests confirmed that all data satisfied the assumptions of normality. Two-way repeated-measures analysis of variance (ANOVA) was used to assess the effects of frequency and the side of the legs on CoP_ap_ speed. For the CoP_ap_ speed, when significant interactions between frequency and leg side were recognized, the *post hoc* Tukey’s honestly significant difference (HSD) test and Bonferroni-adjusted paired *t*-test were used to investigate differences within each factor, respectively. For all analyses, Greenhouse-Geisser adjustments to the degrees of freedom were applied where appropriate. Pearson correlation coefficients between the lateral differences in CoP_ap_ speed in the no-oscillation condition and in each oscillation condition were used to assess the changes in dominant side of postural stability at the individual level between static and dynamic balance condition. The χ^2^ goodness-of-fit test was used to study the number of subjects in each quadrant for the correlations between the no-oscillation and oscillation conditions and the concordance rate between the dominant side of stability and footedness. One-way repeated-measures ANOVA was used to compare the amplitude of the frequency spectrum across conditions. *Post hoc* analysis was performed using Tukey’s HSD test. The α-level was set at *P* < 0.05. All statistical analyses were performed using IBM SPSS Statistics version 21 software (IBM Japan, Tokyo, Japan).

## Results

According to the results of the Waterloo Footedness Questionnaire–Revised, 85.7% (*n* = 26), 14.3% (*n* = 4) and 0% (*n* = 0) of subjects exhibited right-footedness, mixed-footedness and left-footedness, respectively. The proportion of right-footedness was higher than that of mixed-footedness (χ^2^_ 2_ = 39.2, *P* < 0.001).

**Figure 2:**
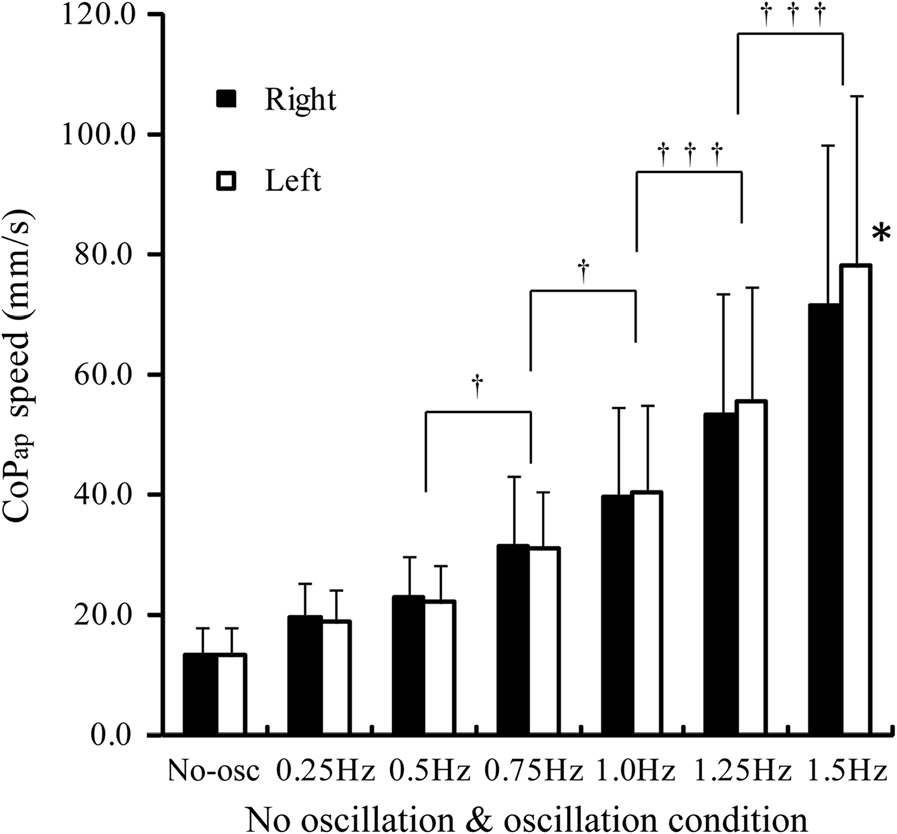
**Mean center of pressure in anteroposterior direction speed during right- and left-leg stance in the no-oscillation and oscillation conditions.** Asterisks indicate significant differences between right- and left-leg stances. Daggers indicate significant differences between adjacent oscillation conditions in each leg. CoP_ap_, Center of pressure in anteroposterior direction; No-osc, No-oscillation condition. *P < 0.05; ^†^*P* < 0.05; ^†††^*P* < 0.001.

CoP_ap_ speeds for right- and left-leg stance in each condition are presented in Figure 2. A significant interaction between the oscillation frequency and leg side were observed (frequency × side: *F*_6,174_ = 6.53; *P*s < 0.05). CoP_ap_ speed exponentially increased with oscillation frequency and showed significant differences between adjacent oscillation conditions with oscillation >0.5 Hz (*P*s < 0.05). For the no-oscillation condition and oscillation frequencies ≤1.25 Hz, no significant difference between the right- and left-leg stances was observed in association with CoP_ap_ speed. For oscillation at 1.5 Hz, CoP_ap_ speed was smaller for right-leg stance than for left-leg stance (*t*_29_ = 3.58, *P* < 0.05).

**Figure 3:**
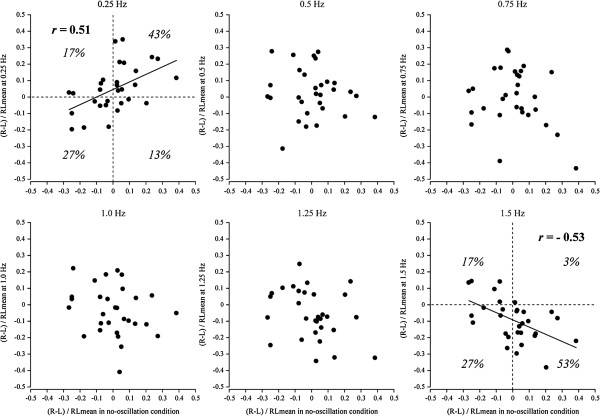
**Relation between the lateral difference in center of pressure in anteroposterior direction speed in the no-oscillation condition and that in the oscillation condition.** L: Left leg; R: Right leg; RLmean: Mean value of center of pressure in anteroposterior direction speed in the left and right legs.

Figure 3 shows the relation between the lateral difference in CoP_ap_ speed in the no-oscillation condition and each oscillation condition. The lateral difference in postural stability in the no-oscillation condition was positively correlated with the lateral difference in postural stability at 0.25-Hz oscillation (*r* = 0.51, *P* < 0.05; *y* = 0.459*x* + 0.046) and negatively correlated with the lateral difference in postural stability at 1.5-Hz oscillation (*r* = -0.53, *P* < 0.05; *y* = -0.439*x* - 0.091). No significant correlation was found for the other oscillation frequencies. Table 1 shows the number of subjects in each quadrant in Figure 3. At 0.25 Hz, the proportions of subjects with right- and left-side dominance were 40% and 60%, respectively, whereas at 1.5 Hz, the proportions of subjects were 80% and 20%, respectively. These proportions of subjects between 0.25 Hz and 1.5 Hz were significantly different (χ^2^_1_ = 33.3, *P* < 0.001). For 70% of subjects, the dominant side of the stability at 0.25-Hz oscillation was equivalent to that at no oscillation (first quadrant: 43.3%; third quadrant: 26.7%), but the dominant side at 1.5-Hz oscillation was different from that at no oscillation (second quadrant: 16.7%; fourth quadrant: 53.3%). Ninety-four percent of subjects with left-side dominance at no oscillation changed to right-side dominance at 1.5-Hz oscillation (fourth quadrant/first and fourth quadrants), whereas only 38% of subjects with right-side dominance at no oscillation changed to left-side dominance at 1.5-Hz oscillation (second quadrant/second and third quadrant). These percentages were significantly different (χ^2^_ 1_ = 4.89, *P* < 0.05). In the no-oscillation condition, the proportion of subjects with concordance between the dominant side of postural stability and the dominant side of mobilizing actions was 50.0%. In the oscillation condition, the proportions were 46.7%, 40.0%, 53.3%, 53.3%, 53.3% and 73.3% for oscillations at 0.25, 0.5, 0.75, 1.0, 1.25 and 1.5 Hz, respectively. The concordance rate at 1.5-Hz oscillation was larger than at the other frequencies (χ^2^_ 1_ = 6.53, *P*s < 0.05).

**Table 1:** **Number of subjects in each quadrant of correlation diagram in Figure**3

			**Dynamic condition**											
	**Dominant side**		**0.25 Hz**		**0.5 Hz**		**0.75 Hz**		**1.0 Hz**		**1.25 Hz**		**1.5 Hz**	
Quadrant	Static-dynamic		*n*	%	*n*	%	*n*	%	*n*	%	*n*	%	*n*	%
First	Left	Left	13	43.3	12	40.0	9	30.0	5	16.7	3	10.0	1	3.3
Second	Right	Left	5	16.7	6	20.0	7	23.3	7	23.3	9	30.0	5	16.7
Third	Right	Right	8	26.7	7	23.3	6	20.0	6	20.0	4	13.3	8	26.7
Fourth	Left	Right	4	13.3	5	16.7	8	26.7	12	40.0	14	46.7	16	53.3

Static: No-oscillation condition; Dynamic: Oscillation condition. Left: Left side dominance in postural stability. Right: Right side dominance in postural stability.

**Figure 4:**
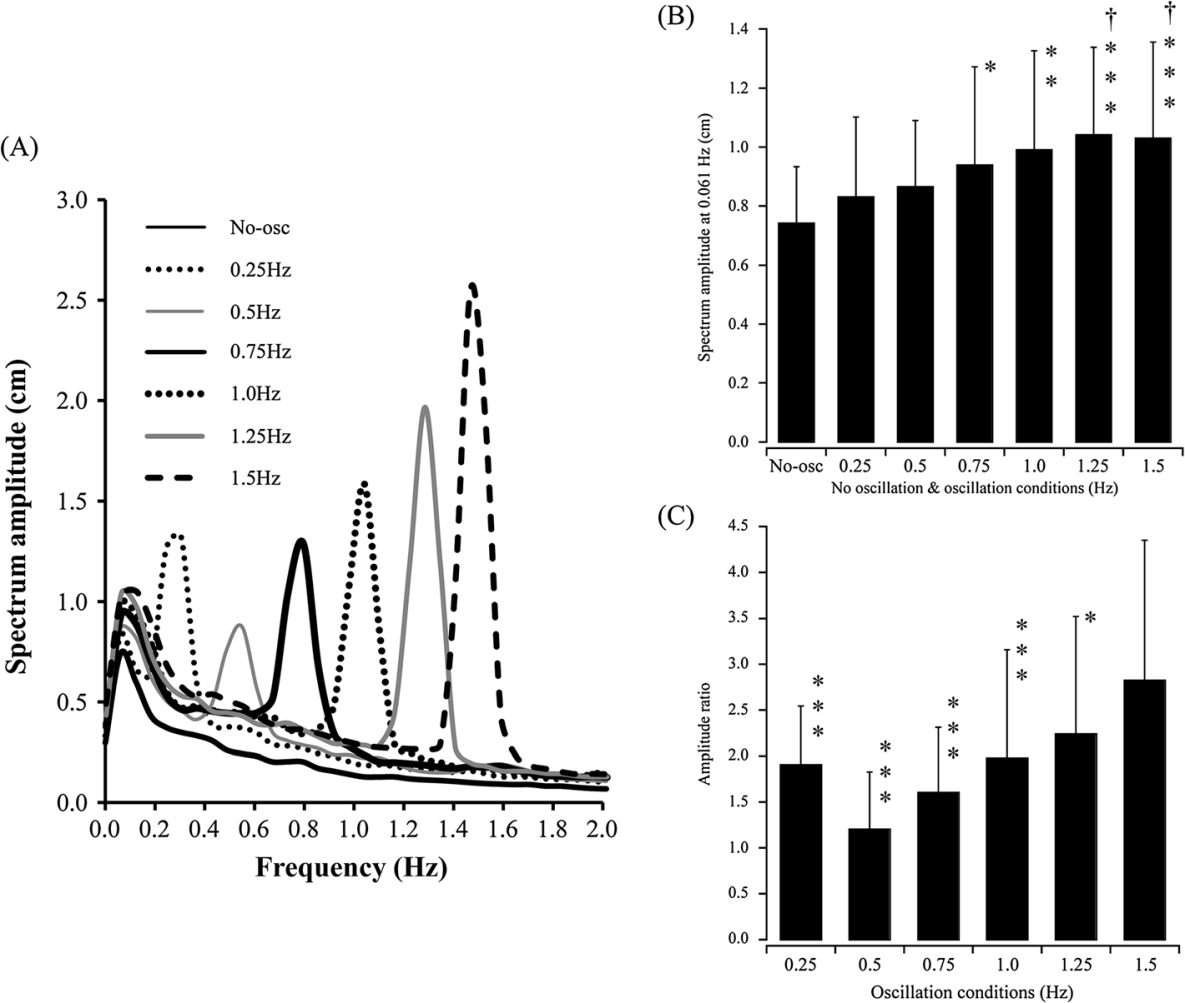
**Frequency analysis of center of pressure in anteroposterior direction sway. (A)** Grand average waveforms of the frequency spectrum of center of pressure in anteroposterior direction (CoP_ap_) sway. The frequency spectrum was averaged across right- and left-leg stances. No osc: No-oscillation condition. **(B)** Amplitude of the low-frequency peak of the frequency spectrum in no-oscillation and oscillation conditions. Asterisks and daggers indicate significant differences relative to no oscillation and 0.25 Hz oscillation, respectively. *P < 0.05, **P < 0.01, ***P < 0.001, ^†^*P* < 0.05. **(C)** Ratio of the amplitude of frequency spectrum at oscillation frequency to that at 0.061 Hz. Asterisks indicate significant differences relative to 1.5 Hz. *P < 0.05, ***P < 0.001.

There was no significant difference between the peak frequency of CoP_ap_ sway for right- and left-side stance in any oscillation frequencies. Figure 4A shows mean spectrum averaged across both sides. In the no-oscillation conditions, the spectrum peak of the low-frequency component occurred at 0.061 Hz (amplitude: 0.74 cm) and the amplitude of spectrum peak was decreased by half at 0.244 Hz. All subjects showed the spectrum peak at <0.3 Hz. Also, at all oscillation frequencies, the spectrum peak was found at the oscillation frequency and the low-frequency component the same as the no-oscillation condition. The amplitude of spectrum peak at 0.061 Hz was significantly larger at 1.0-, 1.25- and 1.5-Hz oscillation than at the no-oscillation condition (*F*_6,14_ = 6.78, *P*s < 0.001), whereas no significant differences between adjacent oscillation conditions were observed with oscillations >0.5 Hz (Figure 4B). Next, in order to compare the spectrum peak at the oscillation frequency between lower- and higher-oscillation conditions, the ratio of the peak amplitude at the oscillation frequency to the peak amplitude at 0.061 Hz was calculated. The ratio of amplitude was significantly higher at 0.25-Hz oscillation than at 0.5-Hz oscillation, and, at >0.5 Hz, it significantly increased with increases in oscillation frequency (*F*_2.2, 64.0_ = 13.5, *P*s < 0.001) (Figure 4C). The ratio at 1.5-Hz oscillation was significantly larger than that at 1.25 Hz (*P* < 0.05).

## Discussion

The results of the present study suggest that the lateral dominance of postural stability during single-leg stance changed according to the frequency of the floor oscillation. Here we discuss the effects of the oscillation frequency on the lateral dominance of postural stability during single-leg stance on the oscillating floor.

At 0.25-Hz oscillation, 70% of the subjects showed that the most stable side was same as that at no oscillation, whereas no significant lateral difference in postural stability was observed. These results indicate that, similar to the static balance condition, no significant lateral difference was shown with low-frequency oscillation, whereas, on an individual level, the dominant side of postural stability was the same as that in static balance, which is consistent with our first hypothesis, described in the Introduction. Fujiwara *et al*. [16] reported that there was no significant difference in postural muscle activity between bipedal stance on a floor oscillating at 0.1 Hz and a stable nonoscillating floor [16]. Buchanan and Horak [22] reported that when the floor oscillated at a low frequency (0.1 or 0.25 Hz), subjects exhibited little damping of head and trunk anteroposterior motion and that anteroposterior center of mass displacement was approximately equal to platform displacement, regardless of whether the eyes were open or closed [22]. These findings indicate control of postural sway at 0.25-Hz oscillation in common with that in static condition. In the present study, common lateral dominance thus would not be observed at 0.25-Hz oscillation, the same as the no-oscillation condition. Spectrum analysis showed that the peak frequency in CoP_ap_ sway was 0.061 Hz in the no-oscillation condition and that this peak amplitude did not differ between the no-oscillation and 0.25-Hz oscillation conditions. Even though 0.25-Hz oscillation was the smallest acceleration stimulus among oscillation conditions, the ratio of peak amplitude at the oscillation frequency to that at 0.061 Hz was larger in the 0.25-Hz condition than in the 0.5-Hz condition (Figure 4C). In 0.25-Hz oscillation, the low-frequency component overlapped with the component of oscillation frequency. These results of our present study indicate that body sway during single-leg stance at 0.25-Hz oscillation was similar to body sway at no oscillation, which relates to the consistency in the dominant side of postural stability between the 0.25-Hz and no-oscillation conditions.

At 1.5-Hz oscillation, however, 80% of subjects showed right-side dominance of postural stability, and a negative correlation was found between lateral differences in no-oscillation and oscillation conditions at this frequency. In addition, 85% of subjects showed right-side dominance of mobilizing functions, and the proportion of subjects with concordance between the dominant side of postural stability and mobilizing functions was high as 73%. These results support our second hypothesis described in the Introduction that, with increases of oscillation frequency, the postural stability of the dominant side for mobilizing functions would become greater than that of the nondominant side. In previous studies of dynamic balance during arm movements [23] and stepping [17], anticipatory activation of postural muscles in the supporting foot was different between the left and the right sides. Additionally, the dominant side of mobilizing function could diminish the disturbance efficiently and thus had high postural stability. Therefore, the main factor related to the lateral dominance of postural stability in the dynamic condition would be the lateral dominance of mobilization. However, in our present study, 38% of subjects with right-side dominance of postural stability in the no-oscillation condition changed their dominant side at 1.5-Hz oscillation. This suggests that not only mobilizing functions but also specialization of support function between left and right legs would affect the difference of postural control during single-leg stance between static and dynamic balance conditions.

It is possible that the right-side dominance for postural stability in dynamic balance condition is related to the functional lateral dominance between the left and right hemispheres. With regard to the laterality of the cerebral hemispheres, functional differences between the hemispheres are not found at low processing levels, but are clearly found at higher processing levels [24]. The central nervous system for postural control is divided into reflex- and situation-dependent adaptation [25]. The former is composed primarily of the brainstem, spinal cord and cerebellum and is closely involved in the control of static balance [26,27]. The latter also incorporates the diencephalon, basal ganglia and cerebral cortex and is involved in the control of dynamic balance. The right hemisphere is dominantly involved in spatial perception and attentional function directed to somatosensory information, whereas the left hemisphere is dominantly involved in the cognition of sound duration and continuity and rhythm [28]. During periodic floor oscillation, the anticipation of the regular postural disturbance is essential; thus, the left hemisphere may play a relatively important role.

In the oscillations from 0.5 to 1.25 Hz, no lateral dominance in the postural stability was observed. In addition, the ratio of amplitude of the frequency spectrum at the oscillation frequency to the amplitude of the frequency spectrum at 0.061 Hz significantly increased with oscillation frequencies over 0.5 Hz and significantly differed between oscillations at 1.25 and 1.5 Hz. The results of previous studies of the frequency spectrum of body sway during bipedal standing suggest that low-frequency components from 0 to 0.5 Hz would reflect the postural control by visual–vestibular system [20,21], but high-frequency components >0.5 Hz by proprioception [21,29]. Therefore, during high-frequency floor oscillation, the contribution of the visual system to postural control would be constant, but that of proprioception would increase from 1.25 to 1.5 Hz. It has been reported that the soleus showed continuous activation for maintenance of posture in no-oscillation or low-frequency floor oscillation, but burst activation for dynamic postural control in high-frequency floor oscillation [16]. This would indicate that, in the 1.5-Hz condition, the dynamic elements for postural control would be strongly required to maintain postural stability. During repetitive movements such as hopping [30,31], skipping rope [32] and free walking [33], efficient and stable motor control was observed when the movement was performed at relatively higher frequencies ≥1.5 Hz. Therefore, lateral dominance in postural stability may be observed in floor oscillations at frequencies >1.5 Hz, which may clarify the border of frequency for stability of dynamic postural control in single-leg stance. In future studies, we will address the lateral difference in postural stability at frequencies >1.5 Hz.

## Conclusion

In static conditions, there was no lateral dominance of stability during single-leg stance. At 1.5-Hz oscillation, the highest frequency, right-side dominance of postural stability was recognized. The functional role in the supporting leg may be divided between the left and right legs according to the change in balance condition from static to dynamic.

## Competing interests

The authors declare that they have no competing interests.

## Authors’ contributions

TK and KF developed the idea for the study, planned the methods, directed the experiments, interpreted the results and drafted the manuscript. Both authors read and approved the final manuscript.
